# Antitumor Effect of Sclerostin against Osteosarcoma

**DOI:** 10.3390/cancers13236015

**Published:** 2021-11-29

**Authors:** Hirokazu Ideta, Kazushige Yoshida, Masanori Okamoto, Jun Sasaki, Munehisa Kito, Kaoru Aoki, Yasuo Yoshimura, Shuichiro Suzuki, Atsushi Tanaka, Akira Takazawa, Hisao Haniu, Takeshi Uemura, Takashi Takizawa, Atsushi Sobajima, Takayuki Kamanaka, Jun Takahashi, Hiroyuki Kato, Naoto Saito

**Affiliations:** 1 Department of Orthopaedic Surgery, Shinshu University School of Medicine, Matsumoto 390-8621, Japan; ideta@shinshu-u.ac.jp (H.I.); kazuyoshi@shinshu-u.ac.jp (K.Y.); junjun_panda2003@yahoo.co.jp (J.S.); mune0527@yahoo.co.jp (M.K.); kin29men@shinshu-u.ac.jp (K.A.); ss5456@shinshu-u.ac.jp (S.S.); tanaatsu@shinshu-u.ac.jp (A.T.); takashitak@shinshu-u.ac.jp (T.T.); soba@shinshu-u.ac.jp (A.S.); kam17@shinshu-u.ac.jp (T.K.); jtaka@shinshu-u.ac.jp (J.T.); hirokato@shinshu-u.ac.jp (H.K.); 2 Department of Applied Physical Therapy, Shinshu University School of Health Sciences, Matsumoto 390-8621, Japan; 3 Department of Orthopedic Surgery, Shinshu Ueda Medical Center, Ueda 386-8610, Japan; yyoshim@shinshu-u.ac.jp (Y.Y.); takazawa@shinshu-u.ac.jp (A.T.); 4 Institute for Biomedical Sciences, Interdisciplinary Cluster for Cutting Edge Research, Shinshu University, Matsumoto 390-8621, Japan; hhaniu@shinshu-u.ac.jp (H.H.); tuemura@shinshu-u.ac.jp (T.U.); saitoko@shinshu-u.ac.jp (N.S.)

**Keywords:** osteosarcoma, sclerostin, Wnt pathway

## Abstract

**Simple Summary:**

Osteosarcoma is highly variable and heterogeneous, which is one of the reasons for its resistance to treatment. Because osteosarcoma is defined by abnormal bone formation, we hypothesize its suppression could lead to effective treatment for all types of osteosarcomas. Sclerostin is secreted by osteocytes and inhibits the canonical pathway by binding to LRP5/6, thereby suppressing bone formation. The resulting suppression of bone formation leads to bone loss and osteoporosis. Here, we investigated the antitumor effect of sclerostin against osteosarcoma and found that sclerostin suppressed the proliferative capacity and migratory ability of osteosarcoma cells.

**Abstract:**

Various risk factors and causative genes of osteosarcoma have been reported in the literature; however, its etiology remains largely unknown. Bone formation is a shared phenomenon in all types of osteosarcomas, and sclerostin is an extracellular soluble factor secreted by osteocytes that prevents bone formation by inhibiting the Wnt signaling pathway. We aimed to investigate the antitumor effect of sclerostin against osteosarcoma. Osteosarcoma model mice were prepared by transplantation into the dorsal region of C3H/He and BALB/c-nu/nu mice using osteosarcoma cell lines LM8 (murine) and 143B (human), respectively. Cell proliferations were evaluated by using alamarBlue and scratch assays. The migratory ability of the cells was evaluated using a migration assay. Sclerostin was injected intraperitoneally for 7 days to examine the suppression of tumor size and extension of survival. The administration of sclerostin to osteosarcoma cells significantly inhibited the growth and migratory ability of osteosarcoma cells. Kaplan–Meier curves and survival data demonstrated that sclerostin significantly inhibited tumor growth and improved survival. Sclerostin suppressed the proliferative capacity and migratory ability of osteosarcoma cells. Osteosarcoma model mice inhibited tumor growth and prolonged survival periods by the administration of sclerostin. The effect of existing anticancer drugs such as doxorubicin should be investigated for future clinical applications.

## 1. Introduction

Osteosarcoma is a malignant tumor derived from osteoblast lineage cells. The tumor forms the neoplasm of bone and osteoid [1,2]. The spectrum of the disease is broad, and its etiology is often unknown. There have been many reports about its risk factors (age, height, race, radiation, bone diseases, and/or hereditary cancer syndrome) and responsible genes [3,4,5,6,7,8,9,10,11,12,13]. Osteosarcoma is highly diverse and heterogeneous, which is one of the reasons for its resistance to treatment.

Osteosarcoma is treated with a combination of surgery and multidrug chemotherapy, including methotrexate, doxorubicin, and cisplatin. Neoadjuvant chemotherapy was introduced in the 1970s and improved the 5-year survival rate to approximately 70%. However, new and effective drugs have not been developed over the past decades, and the survival rate has reached a plateau [14,15].

The Wnt pathway regulates a wide range of phenomena, including homeostasis and the development, growth, differentiation, and maintenance of stem cells. The pathway is conserved in many animal species [16,17]. The canonical pathway is mediated by beta-catenin, while the noncanonical pathway is not. In the canonical pathway, the Wnt ligand binds to the Frizzled and LRP5/6 receptor complex, which transmits signals into the cells. Once β-catenin is accumulated in the cell and transferred to the nucleus, it regulates the expression of target genes, cell proliferation, and differentiation. An abnormally upregulated canonical pathway promotes tumorigenesis and metastasis [18] in addition to the proliferation and differentiation of osteoblasts in bone metabolism [19,20,21]. The noncanonical Wnt pathway has also been reported to indirectly promote bone formation [17].

Sclerostin is mainly secreted by osteocytes and inhibits the canonical pathway by binding to LRP5/6, thereby suppressing bone formation [22]. The resulting suppression of bone formation leads to bone loss and osteoporosis. Mutations in the sclerostin gene can result in conditions associated with high bone mass such as sclerosteosis and van Buchem disease, which are autosomal recessive skeletal diseases characterized by the overgrowth of bone [23,24]. Sclerostin is stimulated by calcitonin [25] and suppressed by parathyroid hormone, mechanical stimulation, and estrogen [26,27,28,29,30,31].

Anti-sclerostin antibody suppresses the effect of bone loss and promotes canonical Wnt pathway signaling and bone formation. This antibody-mediated blockade of sclerostin has been used in many clinical trials for osteoporosis [32,33,34]. However, osteogenic drugs such as parathyroid hormone injections, which activate Wnt signaling by downregulating sclerostin, are suspected of increasing the risk of osteosarcoma due to the excessive bone formation caused by their long duration of treatment in animal experiments [35,36]. We hypothesized that sclerostin would prevent the growth and metastasis of osteosarcoma, which is characterized by abnormal bone formation by inhibiting the canonical Wnt pathway in osteosarcoma cells. This study aims to examine the antitumor effect of sclerostin on osteosarcoma.

## 2. Materials and Methods

### 2.1. Cell Cultures and Reagents

Murine osteosarcoma cell line LM8 (Riken Cell Bank, Tokyo, Japan, RCB Cat# RCB1450, RRID: CVCL_6669) was cultured in α-MEM containing 10% fetal bovine serum (FBS). Human osteosarcoma cell line 143B (Riken Cell Bank, RCB Cat# RCB0701, RRID: CVCL_9W36) was cultured in Dulbecco’s Modified Eagle Medium (DMEM) containing 5% FBS. All cell cultures were maintained in 5% CO_2_ at 37 °C. Recombinant mouse and human SOST/Sclerostin protein (Cat# 1589-ST and 1406-ST) and Wnt3a protein (Cat# 1324-WN and 5036-WN) were purchased from R&D Systems Co. Ltd. (Minneapolis, MN, USA).

### 2.2. Alarmarblue Assay

143B and LM8 cells were treated with 100 ng/mL sclerostin for 3 days. For the control group, the same amount of sterile PBS (phosphate-buffered saline) was added instead of sclerostin. The cells were collected with trypsin. One thousand cells/well were seeded in 96-well plates, adhered for 4 h, and alamarBlue reagent (Bio-Rad Laboratories, Irvine, CA, USA) was subsequently added at 10 µL/well. As a negative control, wells were prepared without seeding cells and adding alamarBlue reagent in the medium (*n* = 3). The fluorescence intensity was measured after 3 h according to the manual. The mean value of the negative control was subtracted from the measured value to make the correction.

### 2.3. Scratch Assay

143B and LM8 cells were seeded on 24-well plates. The cells were treated with 100 ng/mL sclerostin for 3 days until reaching 100% confluence. Cell monolayers were scratched using a 200 µL pipette tip. The medium was changed to remove cell debris, and 100 ng/mL sclerostin was subsequently added to the treated group. For the control group, the same amount of sterile PBS was added instead of sclerostin. Images were taken at 0, 4, and 16 h after scratch. ImageJ software was used to quantify the area of the scratched region.

### 2.4. Migration Assay

Cell migration ability was investigated with transwell chamber kits with an 8 μm pore size polycarbonate membrane (Corning, New York, NY, USA). 143B and LM8 cells were treated with sclerostin at 200 and 100 ng/mL, respectively, for 3 days. For the control group, cells were cultured without sclerostin. A total of 5 × 10^4^ 143B and 4 × 10^4^ LM8 cells in 100 µL serum-free medium were seeded in the upper chamber. A 650 µL medium containing 10% FBS was added to the bottom chamber. 143B and LM8 cells were incubated at 37 °C in 5% CO_2_ for 4 and 16 h, respectively. Non-migrated cells on the upper side of the membrane were removed with a cotton swab. Cells on the lower surface of the membrane were fixed and stained with a Diff-Quik staining kit (Sysmex Corporation, Kobe, Japan). Migrated cells were counted with an optical microscope in the five randomly selected areas.

### 2.5. Mice

Mice were housed and maintained at the Committee for Animal Experiments of Shinshu University. Based on national regulations and guidelines, all experimental procedures were reviewed by the Committee for Animal Experiments with final approval by the president of Shinshu University. The animal protocol was approved by the Committee for Animal Experiments of Shinshu University (Approval No. 280112). Male C3H/HeSlc mice (3 weeks of age) and male BALB/c-nu/nu mice (4 weeks of age) were purchased from Japan SLC (Shizuoka, Japan). Mice that exhibited signs of suffering by tumor growth, feeding disorder, and gait disturbance were euthanized for reaching a humane endpoint.

### 2.6. Administration of Sclerostin on Subcutaneously Transplanted Mouse Model of Osteosarcoma

Murine osteosarcoma cell line LM8 was subcutaneously transplanted into the dorsum of 4-week-old C3H/HeS1c mice (1 × 10^7^ cells per mouse, *n* = 7). Human osteosarcoma cell line 143B was subcutaneously transplanted into 5-week-old BALB/c nu/nu mice (1 × 10^7^ cells per mouse, *n* = 7). Species-matched sclerostin was intraperitoneally administered once a day for 7 days at 80 ng/g of body weight per dose. Two hundred microliters of PBS was intraperitoneally administered to the control group at the same timing of the sclerostin group. Mice were followed up until reaching the humane endpoint or natural death. Changes in macroscopic tumor size, body weight, and micro-CT imaging of tumor and lung were observed over time.

### 2.7. Western Blotting

143B and LM8 cells were seeded in a 24-well plate with 100 ng/mL sclerostin and harvested after 72 h incubation. For the control group, cells were cultured without sclerostin. Cells were washed with PBS and collected by a cell scraper. Then, cells were lysed by sonication in a RIPA buffer with a protease inhibitor. The supernatant was harvested after centrifugation at 12,577 × *g* for 30 min. Total protein was quantified using a BCA protein assay kit (Thermo Fisher Scientific Inc., Waltham, MA, USA).

Proteins were separated by SDS-PAGE with 3 µg of protein loaded on each sample and were transferred onto PVDF membranes (Bio-Rad Laboratories, Irvine, CA, USA). The membranes were blocked using the PVDF Blocking Reagent for Can Get Signal^®^ Immunoreaction Enhancer Solution (Toyobo Co., Ltd., Osaka, Japan) for 1 h at RT. The membranes were incubated with anti-β-catenin primary antibody (1:5000 143B, 1:1000 LM8) overnight at 4 °C. The membranes were incubated with secondary antibody conjugated to horseradish peroxidase (1:25,000) for 1 h at RT. Can Get Signal^®^ Immunoreaction Enhancer Solution (Toyobo Co., Ltd., Osaka, Japan) was used for dilution of antibodies. The proteins were visualized by ECL select Western blotting detection reagent (GE Healthcare, Chicago, IL, USA) and were quantified using the ImageQuant TL image analysis software (GE Healthcare, Chicago, IL, USA). Intensities of β-actin signals were normalized with those of β-actin signals. All the whole western blot figures can be found in the supplementary materials.

### 2.8. Statistical Analysis

Statistical analysis was performed with SPSS software ver. 25 (IBM, Armonk, NY, USA) using the unpaired two-tailed *t*-test or Bonferroni’s multiple comparison test. Kaplan–Meier curves were created by the same software, and log rank analysis was used for comparison between groups. *p* values less than 0.05 were considered statistically significant.

## 3. Results

### 3.1. Sclerostin Decreases β-Catenin Expression

**Figure 1:**
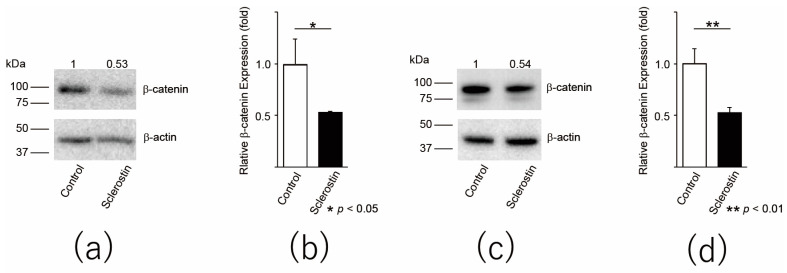
Sclerostin decreases the expression level of β-catenin in osteosarcoma cells. (**a**) The protein expression of β-catenin in LM8 murine osteosarcoma cell line was detected by Western blot assay; (**b**) Quantification of β-catenin signals of (**a**); (**c**) The protein expression of β-catenin in 143B cell line was detected by Western blot assay; (**d**) Quantification of β-catenin signals of (**c**). * *p* < 0.05, ** *p* < 0.01.

In order to confirm the effect of sclerostin on the Wnt pathway of osteosarcoma, we examined the expression level of β-catenin in LM8 and 143B by Western blot assay. The protein expression was decreased in the cells treated with sclerostin (LM8: *p* = 0.03, Figure 1a,b; 143B: *p* = 0.006, Figure 1c,d). These results indicated that sclerostin inhibits the Wnt pathway in osteosarcoma cell lines. For the original Western blots, see Supplementary Figure S1.

### 3.2. Sclerostin Inhibits Proliferation and Migration of Murine Osteosarcoma Cell Lines

**Figure 2:**
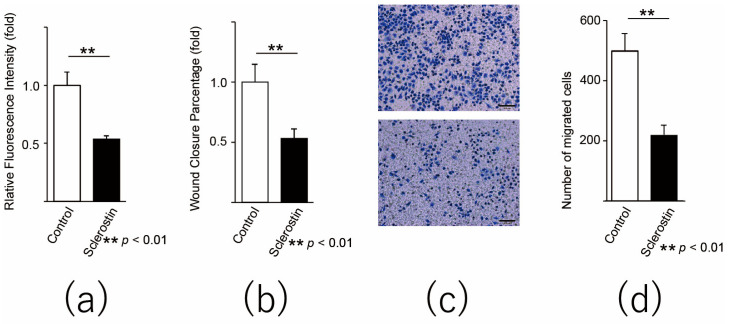
Sclerostin inhibits the proliferation and migration of murine osteosarcoma cell line. (**a**) alamarBlue assay, (**b**) scratch assay, and (**c**,**d**) migration assay using the LM8 murine osteosarcoma cell line. The cells were incubated with sclerostin for 3 days before the experiments. For the scratch assay, sclerostin was added after changing the medium following the scratch procedure. (**c**) The upper image shows the control, and the lower image shows sclerostin-added cells. The bar indicates 100 µm. ** *p* < 0.01.

In order to evaluate the effect of sclerostin on the proliferative potential of the mouse osteosarcoma cell line, an alamarBlue assay was performed. When sclerostin was added to the culture medium of the mouse osteosarcoma cell line LM8, sclerostin significantly inhibited the growth of LM8 (*p* < 0.001, *p* = 0.0000018, Figure 2a). In addition, a scratch assay was performed to clarify the effects of sclerostin on the proliferation and migration of mouse osteosarcoma cell lines. When sclerostin was added to LM8, the reduction in the wound area was significantly delayed (*p* < 0.001, *p* = 0.0089, Figure 2b). The results suggest that sclerostin inhibits not only the proliferation but also the migration of mouse osteosarcoma cells.

Next, to analyze the effect of sclerostin on the migration ability of the mouse osteosarcoma cell line, we performed a migration assay and found that sclerostin significantly inhibited the migration of LM8 (*p* = 0.000002, Figure 2c).

### 3.3. Sclerostin Inhibits Proliferation and Migration of Human Osteosarcoma Cell Lines

**Figure 3:**
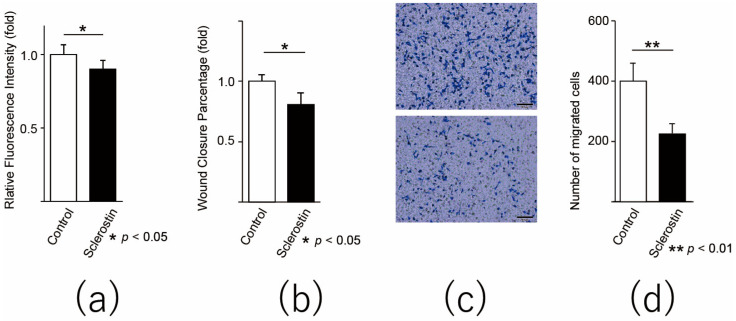
Sclerostin inhibits the proliferation and migration of human osteosarcoma cell line. (**a**) alamarBlue assay, (**b**) scratch assay, and (**c**,**d**) migration assay using the 143B human osteosarcoma cell line. The cells were incubated with sclerostin for 3 days before the experiments. For the scratch assay, sclerostin was added after changing the medium following the scratch procedure. (**c**) The upper image shows the control, and the lower image shows sclerostin-added cells. The bar indicates 100 µm. * *p* < 0.05, ** *p* < 0.01.

We examined whether the inhibitory effects of sclerostin on the proliferation and migration of mouse cell lines can also be observed in human osteosarcoma cell lines. When tested in the alamarBlue assay, the addition of sclerostin to the culture medium of the human osteosarcoma cell line 143B significantly inhibited the growth of the cell line (*p* = 0.040, Figure 3a). Furthermore, in the scratch assay using 143B, the reduction in wound area was significantly delayed in the sclerostin group (*p* = 0.040, Figure 3b). In the migration assay, the exposure of sclerostin significantly inhibited the migration ability of 143B (*p =* 0.009, Figure 3c).

These findings indicate that sclerostin inhibits the activity of the Wnt pathway in addition to proliferative and migratory abilities of mouse and human osteosarcoma cell lines.

### 3.4. Intraperitoneal Administration of Sclerostin Inhibits Tumor Growth and Prolongs Overall Survival in a Mouse Model of Subcutaneously Transplanted Murine Osteosarcoma 

**Figure 4:**
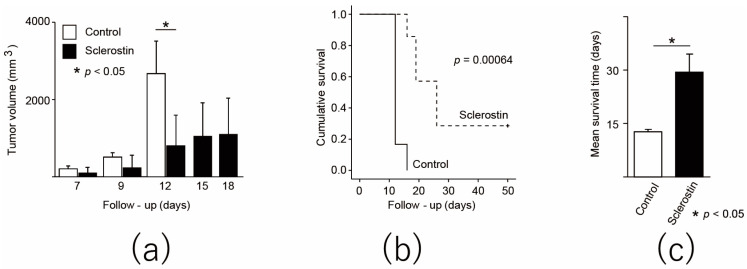
Sclerostin inhibits tumor growth and improves overall survival in mice transplanted with murine osteosarcoma cell line. (**a**) Change in transplanted tumor volume. All control mice met the euthanasia criteria after day 12; (**b**) The Kaplan–Meier curve of control group and sclerostin group; (**c**) Mean survival time from tumor transplantation. * *p* < 0.05.

Sclerostin was administered to mice that were subcutaneously transplanted with the murine osteosarcoma cell line LM8. The mouse models were subsequently evaluated in vivo. Sclerostin was administered once per day for seven days and compared to the control. An increase in tumor volume was suppressed in the sclerostin group, and a significant difference was found at 2 weeks after transplantation (control: 2672.75 ± 838.32 mm^3^, sclerostin: 806.64 ± 787.82 mm^3^, *p* = 0.017, Figure 4a). Survival curves were significantly improved (Figure 4b), and mean survival significantly increased from 12.67 ± 1.63 days in the control group to 29.43 ± 13.54 days in the sclerostin group (n = 7, *p* = 0.018, Figure 4c). No adverse event was observed in the sclerostin group. In a mouse model that was subcutaneously transplanted with a mouse osteosarcoma cell line, sclerostin was found to inhibit tumor growth and prolong overall survival.

### 3.5. Intraperitoneal Administration of Sclerostin Inhibits Tumor Growth and Prolongs Overall Survival in a Mouse Model of Subcutaneously Transplanted Human Osteosarcoma

**Figure 5:**
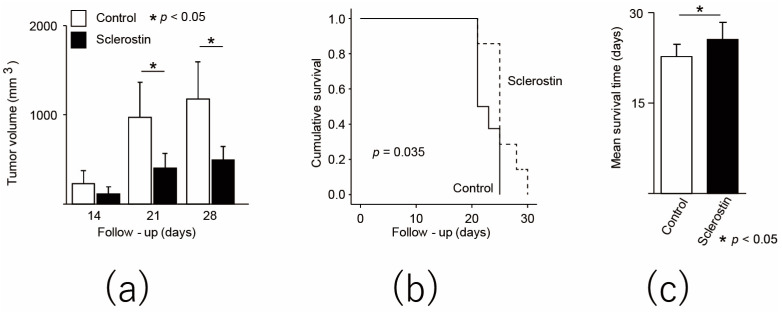
Sclerostin inhibits the tumor growth and improves the overall survival of mice transplanted with human osteosarcoma cell line. (**a**) Change in transplanted tumor volume; (**b**) The Kaplan–Meier curve of the control group and sclerostin group; (**c**) Mean survival time from tumor transplantation. * *p* < 0.05.

Next, human sclerostin was administered to nude mice that were subcutaneously transplanted with human osteosarcoma cell line 143B. The mouse models were then evaluated in vivo. An increase in tumor volume was suppressed in the sclerostin group, and a significant difference was found at 3 weeks after transplantation (control: 972.68 ± 391.52 mm^3^, sclerostin: 399.18 ± 163.17 mm^3^, *p* = 0.0032, Figure 5a). Survival curves were significantly improved (Figure 5b), and mean survival significantly increased from 22.75 ± 1.98 days in the control group to 25.57 ± 2.82 days in the sclerostin group (*n* = 7, *p* = 0.041, Figure 5c). No adverse event was observed in the sclerostin group. In a mouse model that was subcutaneously transplanted with a human osteosarcoma cell line, sclerostin was found to inhibit osteosarcoma growth and prolong overall survival.

## 4. Discussion

In this study, we showed that sclerostin, a canonical Wnt pathway inhibitor, suppresses the proliferation and migration ability of mouse and human osteosarcoma cell lines. Furthermore, we showed that sclerostin inhibited the growth of the tumor and prolonged the survival time in the mouse model of osteosarcoma. The number of mice used in this study was limited (*n* = 7); however, because the results for models transplanted with both human and mouse cell lines showed significant improvement, we could conclude that sclerostin is effective in vivo. Previous reports have shown that the canonical Wnt pathway is associated with oncogenesis, tumor growth, and metastasis of a variety of malignant tumors, including colorectal cancer [37], noncolorectal gastrointestinal cancer [38], breast cancer [39], and renal cell carcinoma [40]. Various Wnt inhibitors have been applied to the treatment of many malignant tumors by inhibiting the Wnt pathway [41,42,43,44,45,46,47,48,49]. In bone and soft tissue tumors, the Wnt pathway has received more attention for its importance in desmoid-type fibromatosis [50] and synovial sarcoma [51], and the pathway has received attention as a clinical diagnosis marker and therapeutic target. In osteosarcoma, LRP5 expression has also been reported to be associated with decreased event-free survival [52]. Although there have been some reports on the antitumor effects of Wnt inhibitors [26,53,54], none have been clinically applied.

Sclerostin is a protein encoded by the Sost gene [23,55]. Sclerostin binds to the co-receptor LRP5/6 and blocks the binding of Wnt ligand to LRP5/6, thereby inhibiting the Wnt pathway [56]. Its expression has also been found in malignant bone tumors including osteosarcoma [57,58]. Zou et al. reported that sclerostin expression in osteosarcoma was lower than that in osteochondroma or normal osteoblasts, and its knockdown in osteosarcoma cell lines resulted in increased proliferation [59]. These findings indirectly support our study. Sclerostin is secreted by osteocytes and is thought to be specific to bone tissue [22,60,61].

There are several advantages in the clinical application of sclerostin for the treatment of osteosarcoma. First, systemic side effects may be mild with little effect on other organs due to its high specificity to bone tissue. In fact, no significant side effect was observed in this study, and our results were partially supported by an osteocalcin promoter-derived sclerostin overexpression model [62]. Second, sclerostin inhibits osteogenesis, which is a common definition for all osteosarcomas; thus, it may be effective for many patients, even in those with highly diverse osteosarcomas. Third, sclerostin is not a cytotoxic anticancer drug and could potentially be used in combination with existing anticancer drug treatments. There may be additive effects when used in combination with other anticancer agents. Finally, doxorubicin, one of the key drugs for osteosarcoma, is known to enhance the Wnt pathway in osteosarcoma, which results in drug resistance [63,64]. The combined use of the Wnt inhibitor sclerostin may help overcome the drug resistance of doxorubicin.

On the other hand, there are also disadvantages. First, previous studies have reported that serum sclerostin levels are elevated in patients with osteolytic changes in multiple myeloma and bone metastases from breast cancer, and that the administration of anti-sclerostin antibody suppresses bone destruction in model mice [65,66,67,68,69]. In other words, because sclerostin is an inhibitor of bone formation, its administration could exacerbate osteolytic metastases that occur in other cancers. Thus, sclerostin as a novel therapy is intended to be used only for osteosarcoma, which is defined by its bone formation. Future studies on the effect of sclerostin on osteogenic bone metastases are warranted. Second, sclerostin inhibits bone formation and may cause osteoporosis; therefore, it may be necessary to shorten the duration of treatment by combining other anticancer drugs, choose selective arterial injection to allow sclerostin to be locally effective, and combine bone resorption inhibitors. However, we believe the advantages may exceed the possible side effects in patients with progressing osteosarcoma.

## 5. Conclusions

We investigated the effect of sclerostin, a canonical Wnt pathway inhibitor and osteogenesis suppressor, on osteosarcoma. Sclerostin inhibited the proliferation and migration of osteosarcoma cell lines, suppressed tumor growth, and promoted the survival of osteosarcoma-implanted mice. An assessment on the effects of sclerostin in combination with existing antitumor drugs may provide important insights for novel treatment strategies of osteosarcoma and warrants further investigation.

## Data Availability

The data that support the findings of this study are available from the corresponding author, M.O., upon reasonable request.

## Supplementary Materials

The following are available online at https://www.mdpi.com/article/10.3390/cancers13236015/s1, Figure S1: The whole blot on the Western blotting.
